# Epitaxial Core‐Shell Oxide Nanoparticles: First‐Principles Evidence for Increased Activity and Stability of Rutile Catalysts for Acidic Oxygen Evolution

**DOI:** 10.1002/cssc.202200015

**Published:** 2022-04-13

**Authors:** Yonghyuk Lee, Christoph Scheurer, Karsten Reuter

**Affiliations:** ^1^ Department of Chemistry Chair of Theoretical Chemistry and Catalysis Research Center Technische Universität München Lichtenbergstraße 85747 Garching Germany; ^2^ Fritz-Haber-Institut der Max-Planck-Gesellschaft Faradayweg 4–6 14195 Berlin Germany

**Keywords:** ab initio thermodynamics core-shell particles, DFT calculations, electrolysis, oxygen evolution reaction

## Abstract

Due to their high activity and favorable stability in acidic electrolytes, Ir and Ru oxides are primary catalysts for the oxygen evolution reaction (OER) in proton‐exchange membrane (PEM) electrolyzers. For a future large‐scale application, core‐shell nanoparticles are an appealing route to minimize the demand for these precious oxides. Here, we employ first‐principles density‐functional theory (DFT) and ab initio thermodynamics to assess the feasibility of encapsulating a cheap rutile‐structured TiO_2_ core with coherent, monolayer‐thin IrO_2_ or RuO_2_ films. Resulting from a strong directional dependence of adhesion and strain, a wetting tendency is only obtained for some low‐index facets under typical gas‐phase synthesis conditions. Thermodynamic stability in particular of lattice‐matched RuO_2_ films is instead indicated for more oxidizing conditions. Intriguingly, the calculations also predict an enhanced activity and stability of such epitaxial RuO_2_/TiO_2_ core‐shell particles under OER operation.

## Graphical abstract

**Using first‐principles** density‐functional theory calculations combined with ab initio thermodynamics, we introduce a design protocol for RuO_2_‐based core‐shell catalysts which exhibit enhanced stability and activity under oxygen evolution reaction (OER) operating conditions.

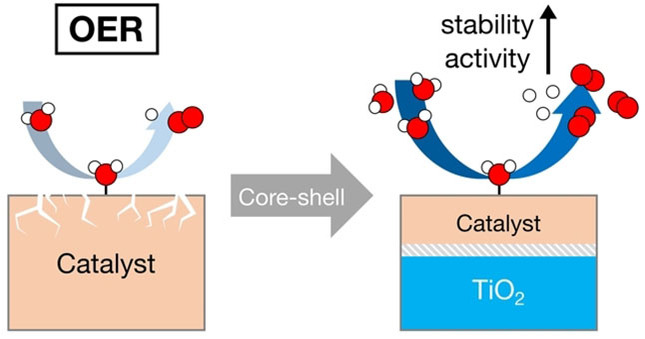

## Introduction

Core‐shell nanoparticle morphologies are a powerful and frequently pursued concept in heterogeneous catalysis to reduce the demand of precious active materials. Proton‐exchange membrane (PEM) water electrolysis^[1]^ is an eminent application area for this concept. Oxides containing rare Ir and Ru are currently the primary anode electrocatalysts for the oxygen evolution reaction (OER) that exhibit both a reasonably small overpotential and sufficient stability under the harsh acidic PEM operating conditions.[^2^, ^3^] Despite the already high efficiency of current generation catalysts, significant further reduction of Ir or Ru mass loading is required when considering that for a prospective hydrogen economy gigantic amounts of electrolysis power will be required.[^3^, ^4^] Within the core‐shell concept, large research efforts have therefore been undertaken towards dispersing the precious active oxides on a variety of inexpensive core materials comprising abundant metals, their nitrides, carbides or oxides.[^5^, ^6^, ^7^, ^8^, ^9^, ^10^] Generally, though, these have been massively loaded composites with incoherent thick Ir or Ru oxide films or small nanoparticles that use the core material more like a high surface‐area support. As one example we highlight IrO_2_ dispersed on TiO_2_,[^11^, ^12^, ^13^] as has also already been commercialized in form of the recent Elyst Ir75 0480 catalyst from Umicore.[^13^, ^14^, ^15^, ^16^, ^17^]

Titanium dioxide exhibits a stable rutile modification. This motivates the idea to instead pursue epitaxial core‐shell nanoparticles with thin coherent films of the equally rutile‐structured IrO_2_ or RuO_2_ enclosing the cheap core material. In this study we explore this idea with detailed first‐principles calculations. Analyzing adhesion, strain and surface energies, we show that prevailing gas‐phase synthesis protocols will only be able to stabilize thin films in the few‐monolayer regime at some low‐index facets of TiO_2_ for both IrO_2_ and RuO_2_. This rationalizes in particular the experimentally observed poor wetting behavior of IrO_2_ at the prevalent (110) facet of rutile TiO_2_ nanoparticles.[^18^, ^19^] Under more oxidizing synthesis conditions, growth of coherent shell films should instead be feasible. Corresponding epitaxial core‐shell particles would obviously minimize the precious metal demand. However, most intriguingly, our ab initio thermodynamics based results additionally indicate an increased stability of such particles under OER operation conditions, as well as an increased activity. At enhanced stability, increased activity and minimized precious metal content, this suggests epitaxial rutile IrO_2_/TiO_2_ or RuO_2_/TiO_2_ core‐shell nanoparticles as a promising target for future synthesis or advanced deposition endeavors.

## Results and Discussion

### Core‐shell interface

As starting point of our investigation we report in Table 1 the computed interface formation energies and work of adhesion for epitaxial and stoichiometric IrO_2_/TiO_2_ and RuO_2_/TiO_2_ interfaces for all five symmetry inequivalent low‐index orientations of rutile, namely (001), (010)/(100), (011)/(101), (110) and (111). In the case of the (111) facet, there are two possible stoichiometric interfaces denoted as t1 and t2, see Figure S1 in the Supporting Information for a description of all interfacial geometries. In the calculation of $\gamma_{interf}^{\left(hkl\right)}$ according to equation (6) we consistently use the optimized TiO_2_ bulk lattice constants in the directions parallel to the interface for all three solid‐state terms which fixes the value of $A^{\left(hkl\right)}$ . This effectively filters out the increasing strain in a coherent MO_2_ film of increasing thickness and allows to arrive at a purely interface specific quantity that reflects the intrinsic cost of creating the interface. Compared to other heterostructures[^20^, ^21^] all calculated interface formation energies are very small (about one order of magnitude smaller than the surface free energies discussed below) and consistently below 30 meV Å^−2^. This shows the expected propensity to form such interfaces between the lattice‐matched oxides.^[22]^ Simultaneously, we always obtain positive values. Bonding between the two materials is thus less favorable than the bonding within the pure materials, and there is not energetic driving force for interdiffusion. This is consistent with experiments reporting an (entropically driven) solid solution of IrO_2_ in TiO_2_ only at temperatures above 900 °C.^[23]^ As complementary key quantities, the negative $W_{adh}^{\left(hkl\right)}$ in Table 1 indicate the energy it would cost to separate the formed interface. Also here, using the optimized TiO_2_ bulk lattice constants in the directions parallel to the interface for all terms entering Equation (7) yields an interface specific quantity that is independent of the thickness of the shell layer. The large negative values obtained for both IrO_2_/TiO_2_ and RuO_2_/TiO_2_ designate a strong intrinsic adhesion, with the same trend over the five low‐index orientations found for both materials: (111) toughest to break and (010)/(100) and (110) offering weakest adhesion. This trend can be rationalized with the number of bonds formed per surface area, which is the number of broken bonds in the unit cell to divide the interface model into two separate TiO_2_ and MO_2_ slabs normalized by the interface area. This trend goes as 0.211, 0.191, 0.160, 0.148 and 0.105 Å^−2^ for (111), (001), (011)/(101), (010)/(100) and (110), respectively. Reformulating $W_{adh}^{\left(hkl\right)}$ from Equation (7) in terms of the interface formation energy and the surface free energies of the stoichiometric TiO_2_ and MO_2_ terminations forming the interface,

(1) $$W_{adh}^{\left(hkl\right)}=\gamma_{interf}^{\left(hkl\right)}-\gamma_{{TiO}_{2},surf}^{\left(hkl\right),stoich}-\gamma_{{MO}_{2},surf}^{\left(hkl\right),stoich},$$

**Table 1:** Calculated interface formation energies $\gamma_{interf}^{\left(hkl\right)}$ and work of adhesion $W_{adh}^{\left(hkl\right)}$ for all five symmetry‐inequivalent low‐index orientations (*hkl*) of epitaxial stoichiometric IrO_2_/TiO_2_ and RuO_2_/TiO_2_ interfaces.

Facet	$\gamma_{interf}^{\left(hkl\right)}$ [meV Å^−2^]		$W_{adh}^{\left(hkl\right)}$ [meV Å^−2^]	
	IrO_2_/TiO_2_	RuO_2_/TiO_2_	IrO_2_/TiO_2_	RuO_2_/TiO_2_
(100)	18	24	−235	−182
(010)/(100)	20	28	−146	−108
(011)/(100)	15	18	−170	−142
(110)	6	23	−130	−101
(111)‐t1	10	16	−253	−216
(111)‐t2	10	15	−254	−217

also allows to trace the consistently stronger adhesion found for the IrO_2_/TiO_2_ interface to the higher respective surface free energies of IrO_2_ as compared to RuO_2_ (see below). Note that at an almost equal cohesive energy of both oxides (computed as −16.95 and −17.16 eV per MO_2_ unit at the present DFT Perdew‐Burke‐Ernzerhof (PBE) functional^[63]^ level, respectively), this difference arises predominantly from a higher polarizability of the larger Ir ion.^[24]^

Consistently offering smaller interface formation energies and stronger adhesion, the analysis up to now would suggest IrO_2_ as preferred shell material. However, the focus on the interface specific quantities $\gamma_{interf}^{\left(hkl\right)}$ and $W_{adh}^{\left(hkl\right)}$ disregards the increasing strain that will build up in pseudomorphic shell layers of increasing thickness. To this end, it is important to realize that the lattice mismatch of RuO_2_−TiO_2_ and IrO_2_−TiO_2_ is quite different for the two main rutile bulk lattice constants, *a* and *c*. Along the longer *a* axis all three materials exhibit almost identical values. With the employed PBE functional this is *a* ${}_{\mathrm{TiO}{}_{2}}$ =4.578 Å and shorter lattice constants by only −1.1 % for both RuO_2_ and IrO_2_. In contrast, along the shorter c axis, this mismatch is larger. Specifically, at PBE level *c* ${}_{\mathrm{TiO}{}_{2}}$ =2.955 Å, while RuO_2_ and IrO_2_ prefer a longer lattice constant by 5.6 % and 7.6 %, respectively. As a consequence of this anisotropy, hardly any strain will build up at the three interface orientations (001), (011)/(101) and (111), whereas much higher strain will build up at the two other orientations (110) and (010)/(100), see the Supporting Information for a detailed account of all values. While the larger strain for IrO_2_ might (partially) scotch the intrinsic advantage of this material in terms of the afore discussed interface specific quantities, we note that much more problematic is the fact that precisely those two orientations that predominantly suffer from strain are those two that offer the weakest intrinsic adhesion anyway, cf. Table 1. This already indicates a high directional dependence of potential core‐shell concepts.

### Surface effects

**Figure 1:**
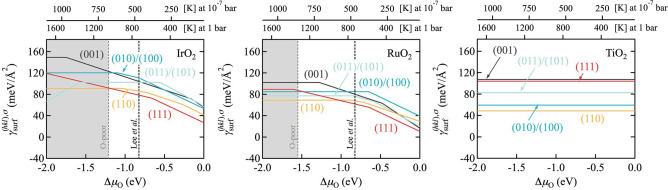
Computed surface free energies $\gamma_{surf}^{\left(hkl\right),\sigma }$ of the five low‐index facets of rutile IrO_2_, RuO_2_ and TiO_2_ in an oxygen environment. Kinks in the individual lines indicate a change of the most stable termination σ, generally going from O‐poor terminations (positive slope with respect to oxygen chemical potential Δ*μ*_O_) over stoichiometric terminations (horizontal lines) to O‐rich terminations (negative slope).[^25^, ^26^, ^27^] The vertical gray dotted lines indicate the thermodynamic bulk oxide stability (O‐poor limit) as computed from the bulk heat of formation (for TiO_2_ this limit is at −3.76 eV outside of the shown range). In the top *x* axis, the dependence on Δ*μ*_O_ is translated into a temperature scale at different oxygen pressures. The black vertical dotted line in the phase diagram for IrO_2_ and RuO_2_ represent the synthesis conditions as employed by Lee et al.^[28]^

For the actual realization of epitaxial core‐shell nanoparticles, not only the interface matters. While growth itself is kinetics, there will always be a thermodynamic driving force to expose the material with the lower surface free energy at the shell. To this end, Figure 1 summarizes the calculated surface free energies of all three oxides, IrO_2_, RuO_2_ and TiO_2_, in an oxygen environment. Equivalent behavior and trends are obtained for the surface free energies in an aqueous environment,^[29]^ cf. the Supporting Information and below, so that the following analysis conceptually also extends to electrodeposition. Explicitly marked in Figure 1 are the synthesis conditions employed by Lee et al. (1 bar, 500 °C)^[28]^ to represent typical gas‐phase synthesis endeavors. For the corresponding range of O chemical potential, Figure 1 immediately reveals a key bottleneck. The surface free energies of IrO_2_ and RuO_2_ are generally not smaller than those of TiO_2_, and in particular for the two orientations (010)/(100) and (110) that were already identified as problematic in terms of their interface stability and strain, TiO_2_ exhibits even significantly lower respective $\gamma_{surf}^{\left(hkl\right),\sigma }$ than the intended shell oxides.

**Figure 2:**
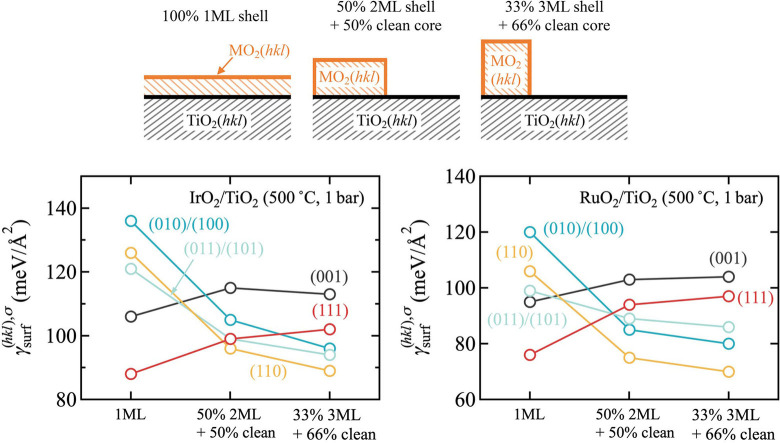
Computed surface free energies $\gamma_{surf}^{\left(hkl\right),\sigma }$ for one monolayer (ML) of shell material pseudomorphically covering the entire TiO_2_(*hkl*) surface, and for two ML (three ML) islands of shell material covering 50 % (33 %) of the TiO_2_(*hkl*) surface with the remaining 50 % (66 %) of the surface uncovered as illustrated in the top panel. Shown is data for all five symmetry‐inequivalent low‐index facets and for IrO_2_ (left panel) and RuO_2_ (right panel) as shell material, respectively. In all cases, the most stable surface terminations σ at the experimental gas‐phase synthesis conditions of Lee et al.^[28]^ were assumed, in accordance with the surface phase diagrams presented in Figure 1.

All aspects, surface, strain and interface, can be combined in a rough estimate of the wetting tendency. As illustrated in Figure 2, this estimate takes the surface free energy $\gamma_{surf}^{\left(hkl\right),\sigma }\left(1\;\;ML\;{MO}_{2}/{TiO}_{2},\;\;100\;\%\right)$ , cf. Equation (9), of a model where a pseudomorphic one monolayer (ML) film of the shell material completely covers the TiO_2_(*hkl*) surface, and compares it to the surface free energy estimates

(2) $$\gamma_{surf}^{\left(hkl\right),\sigma }\left(2\;\;ML\;{MO}_{2}/{TiO}_{2},\;\;50\;\%\right)=\;\frac{1}{2}\left[\gamma_{surf}^{\left(hkl\right),\sigma }\left(2\;\;ML\;{MO}_{2}/{TiO}_{2},\;\;100\;\%\right)+\gamma_{surf}^{\left(hkl\right),\sigma }\left({TiO}_{2},\;\;100\;\%\right)\right]$$

(3) $$\gamma_{surf}^{\left(hkl\right),\sigma }\left(3\;\;ML\;{MO}_{2}/{TiO}_{2},\;\;33\;\%\right)=\;\frac{1}{3}\left[\gamma_{surf}^{\left(hkl\right),\sigma }\left(3\;\;ML\;{MO}_{2}/{TiO}_{2},\;\;100\;\%\right)+{2\gamma }_{surf}^{\left(hkl\right),\sigma }\left({TiO}_{2},\;\;100\;\%\right)\right]$$

Here, $\gamma_{surf}^{\left(hkl\right),\sigma }\left(xML\;{MO}_{2}/{TiO}_{2},\;\;100\;\%\right)$ is the surface free energy of a model where *x*MLs of the shell material pseudomorphically cover the entire TiO_2_(*hkl*) surface and $\gamma_{surf}^{\left(hkl\right),\sigma }\left({TiO}_{2},\;\;100\;\%\right)$ is the surface free energy of the bare TiO_2_(*hkl*) surface. What Equations (2) and (3) thus evaluate is the cost, when the same total amount of shell material is not homogeneously dispersed on the entire surface as in $\gamma_{surf}^{\left(hkl\right),\sigma }\left(1\;\;ML\;{MO}_{2}/{TiO}_{2},\;\;100\;\%\right)$ , but instead forms 2 or 3 ML high islands, respectively, with the remainder of the TiO_2_ surface area uncovered. The simple linear superposition in Equations (2) and (3) thereby disregards any additional costs from the edges of the islands. Hence, $\gamma_{surf}^{\left(hkl\right),\sigma }\left(2\;\;ML\;{MO}_{2}/{TiO}_{2},\;\;50\;\%\right)$ and $\gamma_{surf}^{\left(hkl\right),\sigma }\left(3\;\;ML\;{MO}_{2}/{TiO}_{2},\;\;33\;\%\right)$ represent lower boundaries to the true surface free energies. According to these equations, wetting would require the $\gamma_{surf}^{\left(hkl\right),\sigma }\left(1\;\;ML\;{MO}_{2}/{TiO}_{2},\;\;100\;\%\right)$ of the fully dispersed monolayer to be lower than the surface energies of the two competing island models.

Figure 2 compiles the corresponding data for both IrO_2_ and RuO_2_ as shell materials and using the most stable surface terminations under the synthesis conditions by Lee et al.,^[28]^ cf. Figure 1. Not surprisingly, a wetting tendency is only obtained for the (001) and (111) orientation. These are the orientations with strongest adhesion, minimum strain penalty and comparable or lower MO_2_ surface energies than TiO_2_. The latter lower surface energies result in fact from a qualitatively different behavior of the intended shell oxides that provides an important lead to future synthesis endeavors. Over the entire range of oxygen chemical potential shown in Figure 1, the stoichiometric termination is the most stable termination for all TiO_2_ facets and their surface free energies correspondingly remain constant. In contrast, both shell MO_2_ are able to stabilize O‐rich terminations so that their surface free energies decrease with increasing Δ*μ*_O_. The (001) and (111) facets are able to stabilize such terminations already at lowest oxygen chemical potentials, which is why their surface free energies are already quite low for the synthesis conditions of Lee et al., cf. Figure 1. However, all other facets will eventually also stabilize such terminations, which is why more favorable wetting will generally result for increasingly O‐rich conditions where the MO_2_ surface free energies will continuously decrease.

**Figure 3:**
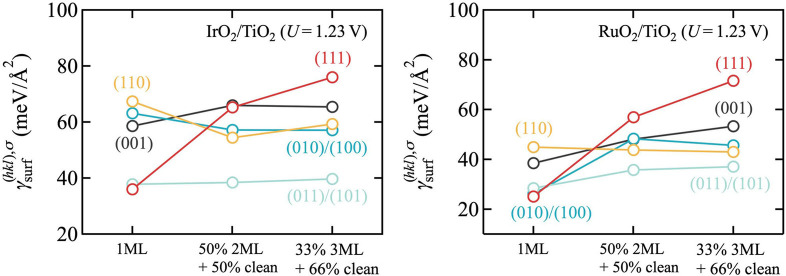
Same as Figure 2, but now computed in an aqueous environment and at the OER equilibrium potential (*U*=1.23 eV), cf. text. A much more favorable wetting tendency is obtained for these more oxidizing conditions, in particular for RuO_2_/TiO_2_.

Unfortunately, for gas‐phase synthesis such conditions are harder to obtain. Most straightforwardly, they would be realized by lowering the temperature, cf. the temperature scales in Figure 1, but then kinetic limitations will increase. Correspondingly, growth has typically been attempted for even less O‐rich conditions than the ones by Lee et al.^[28]^ For instance, in Ref. [19], Abb et al. even used a low oxygen partial pressure of 10^−^7 bar at 700 K. In full agreement with the understanding derived from Figure 1, they found IrO_2_(110) thin films at TiO_2_(110) not to be stable under such conditions. Elevated pressures might instead be a route to achieve more favorable O‐rich conditions in gas‐phase synthesis. However, in light of our results we believe electrodeposition or advanced atomic layer deposition to be more promising routes with easier access to oxidizing conditions.[^30^, ^31^] We illustrate this in Figure 3 with data for the wetting model as in Figure 2, but now computed in an aqueous environment and using the most stable surface terminations in Equation (8) that result at an applied potential corresponding to the OER equilibrium potential (see below). Under these more oxidizing conditions, a much more favorable wetting tendency is obtained. In particular for RuO_2_/TiO_2_, all but the (110) orientation now exhibit a clear preference for wetting. While the (110) orientation is thus certainly the most difficult, we stress that the simple estimates for the 2 or 3 ML island models in Figure 3 disregard any additional costs from the island edges. The corresponding surface free energies should thus be seen as lower bounds to the true surface free energies, and a wetting tendency is almost obtained already when comparing against these lower bounds (see the essentially flat orange line for the (110) facet in Figure 3). In this respect, we tentatively conclude from the present thermodynamic data that growth of epitaxial core‐shell particles should be feasible at sufficiently O‐rich conditions. Importantly, no band gap opening is found in our calculations even for only 1 ML thick films and for all five low‐index facets. This suggests that corresponding particles would also exhibit sufficient electronic conductivity as required for electrocatalytic performance.

### Enhanced stability and activity

**Figure 4:**
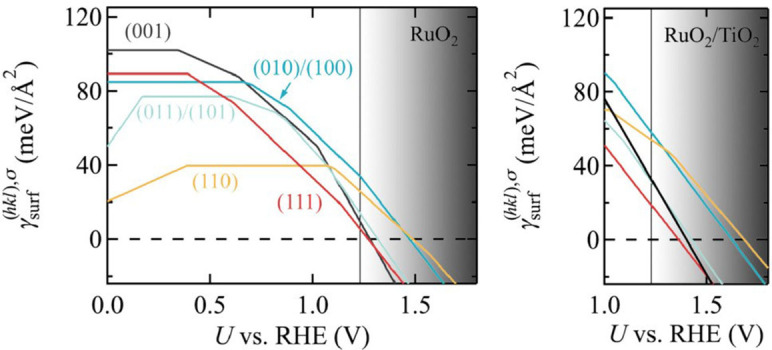
Computed surface free energies $\gamma_{surf}^{\left(hkl\right),\sigma }$ , cf. Equation (8), of the most stable surface terminations σ for all five low‐index facets of rutile RuO_2_ (left panel) and 2 ML RuO_2_/TiO_2_ (right panel) in aqueous environment and as a function of the applied potential *U* from open‐circuit (*U*=0 V vs. RHE) to PEM operating conditions (shaded gray area, taken to be U>1.3 V, see text). The vertical black line indicates the OER equilibrium potential *U*=1.23 V.

Ir and Ru oxides are the primary current OER electrocatalysts. However, even they are known to degrade in the harsh acidic PEM operation conditions, involving a hitherto only incompletely characterized transformation to some amorphous hydrous state.[^2^, ^16^, ^32^, ^33^, ^34^, ^35^, ^36^, ^37^, ^38^, ^39^] In previous work for IrO_2_,^[29]^ we had established a simple thermodynamic descriptor for this degradation. At applied potentials in the OER regime the surface free energies were found to turn negative, indicating a potential thermodynamic instability of the rutile crystal lattice. We here obtain fully analogous results also for RuO_2_ and summarize these findings in Figure 4. As detailed in the Supporting Information, more than 100 terminations *σ* for all five low‐index facets have been computed to systematically consider the different possibilities to adsorb O, H, OH, OH_2_, OOH, and OO species in (1×1) and (1×2) surface unit‐cells. Figure 4 shows the surface free energies of the resulting most stable terminations as a function of the applied potential from open‐circuit (0 V vs. RHE) to conditions relevant for technological PEM operation *U*>1.3 V. The corresponding values at the OER equilibrium potential *U*=1.23 V were also used in the wetting model in Figure 3 above. As in the analogous Figure 1 for gas‐phase conditions, at each facet different terminations become most stable with increasing potential, reflected in Figure 4 by a changing slope of the $\gamma_{surf}^{\left(hkl\right),\sigma }$ line. Generally, we find the expected sequence from fully hydrated or hydroxylated surfaces at open‐circuit conditions to gradually deprotonated terminations at increasing potentials until pure O‐terminations and eventually terminations with higher oxidized superoxo species become most stable at OER‐relevant potentials. A detailed account of these findings and their very good consistency with existing theoretical and experimental data in particular for the best characterized (110) facet is provided in Figure S3 in the Supporting Information.

More relevant for the present context is the pronounced decrease of the surface free energies with increasing potentials. As such, it is in particular $\gamma_{surf}^{\left(111\right),\sigma }$ of the (111) facet that first turns negative already at a critical potential $U_{critical}^{{RuO}_{2}\left(111\right)}$ =1.27 V, that is, just at the OER onset. This reflects a potential thermodynamic instability, as the particle could gain energy by decaying and generating more such surfaces. With the current computational settings and pursuing the exact same approach, for IrO_2_ this critical potential where $\gamma_{surf}^{\left(111\right),\sigma }$ turns negative is computed as $U_{critical}^{{IrO}_{2}\left(111\right)}$ =1.39 V. The concomitantly indicated thermodynamic stability up to higher applied potentials is fully consistent with the well‐established better corrosion resistance of this material[^28^, ^40^, ^41^, ^42^, ^43^, ^44^] and further confirms *U*_critical_ as one of the useful thermodynamic descriptors for evaluating the catalyst stability. Intriguingly, when we compute exactly the same set of surface free energies for a pseudomorphic 2 ML film of RuO_2_ on TiO_2_, we find the decreasing surface free energies of all facets to be shifted toward higher potentials, and thereby obtain higher *U*_critical_ where the surface energies turn negative, cf. Figure 4. In particular, for the (111) facet which is still the one turning negative first, the critical potential is now increased to $U_{critical}^{2ML\;{RuO}_{2}/{TiO}_{2}\left(111\right)}$ =1.36 V, that is, 0.1 V higher than for the pure RuO_2_ particle and close to the corresponding facet of pure IrO_2_. For other facets, this relative increase is up to 0.2 V as detailed in Table S4 in the Supporting Information. According to this thermodynamic descriptor the core‐shell RuO_2_/TiO_2_ particle thus exhibits trends of an increased stability, which is for all facets now essentially the same or better than for a pure IrO_2_ particle.

This striking finding begs the question for its physical origin. Numerically, the shifted *U*_critical_ values result from increased surface free energies of the core‐shell particle compared to those of the native shell oxide, cf. Figure 4. These higher values arise in turn from the additional interface formation energy and strain. Obviously, the shell oxide is not in its optimum state, and if it were for the shell oxide alone, it would be thermodynamically preferable to form a relaxed MO_2_ particle. However, for the entire system a corresponding dewetting or other strain relieving alternatives like extended defects are not competitive as they would necessarily involve the formation of more surface area or expose TiO_2_ with its higher surface free energy at these OER operation conditions. For thicker films this will be changed by the accumulating strain. However, for few ML epitaxial films the increased stability of once formed core‐shell particles results simply out of lack of alternatives and this is how the thermodynamic descriptor should be read.

This cautious reading extends also to another interesting aspect reflected by the calculated surface free energies in Figure 4. The strong decrease in particular of $\gamma_{surf}^{\left(111\right),\sigma }$ with increasing potential as compared to the surface free energies of the other facets shows that it becomes increasingly favorable to form (111) facets. Indeed, combining the fully analogous results for native IrO_2_ within a Wulff construction, we had shown in previous work that the thermodynamically preferred particle shape at OER operation conditions would be one that exclusively exhibits (111) facets.^[29]^ Here, the data summarized in Figure 4 indicates exactly the same thermodynamic driving force to reshape RuO_2_ particles and epitaxial RuO_2_/TiO_2_ particles away from the familiar rutile form with its predominant (110) facets.^[29]^ However, in particular for the core‐shell particle it is uncertain in how much such thermodynamics really applies. To one end, the mechanical hardness of the TiO_2_ core may lead to a very slow kinetics. Additionally, as long as the shell is intact, the TiO_2_ core does not contact the electrolyte and will at least be partially screened from the applied potential. It could thus well be that epitaxial core‐shell particles show an increased resistance to the driving force to reshape – and concomitantly against degradation, either in terms of general mass loss or the fraction of exposed (111) facets with their particularly low *U*_critical_, cf. Figure 4.

We note in passing that CHE is a simplified theoretical approach and that a negative surface energy resulting from such a treatment is not a sound indicator of what detailed physiochemical processes ensue, but merely one readily available stability descriptor which can be used to gauge trends between similar systems. This descriptor neither accounts for the chemical properties of the decomposition products nor for mass transport effects as has been pointed out recently.^[39]^ Such influences can be treated approximately by simple models based on the Poisson‐Nernst‐Planck equations which, to lowest order, will induce shifts in the phase diagrams.^[45]^ For the highly similar systems considered here, we assume that these shifts are approximately constant over the range of systems studied and that our current approach delivers an acceptable semi‐quantitative estimate of which structural modifications might improve the relative stability of the catalyst within its class.

**Figure 5:**
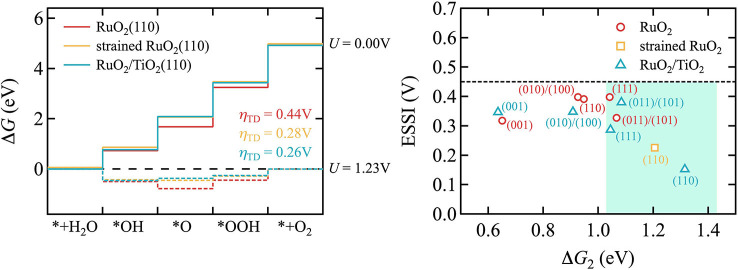
Left panel: Gibbs free energy change along the classic peroxide OER pathway at the (110) surface for pristine RuO_2_ (red), strained RuO_2_ (yellow) and core‐shell 2 ML RuO_2_/TiO_2_ (blue). The four subsequent reaction steps in this pathway are described in the main text. The Illustrated energy profiles are at open‐circuit conditions (*U*=0 V, solid lines) and at the OER equilibrium potential (*U*=1.23 V, dotted lines). In addition, the minimum overpotentials (*η*_TD_) required to make all four steps exergonic are listed. Right panel: The ESSI‐Δ*G*_2_ activity map for pristine RuO_2_ (red), strained RuO_2_(110) (yellow) and core‐shell 2 ML RuO2/TiO2 (blue). The most promising electrocatalysts fall into the highlighted area, which 1.03 eV<Δ*G*_2_<1.43 eV in junction with ESSI<0.45 V.

According to our calculations, epitaxial RuO_2_−TiO_2_ core‐shell particles would not only exhibit an improved stability, but also catalytic activity. Figure 5 demonstrates this by comparing the computed reaction energetics for the prevalent (110) facet for pristine RuO_2_ and for 2 ML RuO_2_−TiO_2_, as well as for RuO_2_ that is equally strained as in the epitaxial core‐shell system. Specifically, this is the reaction energetics along the classic OER peroxide pathway suggested by Rossmeisl et al.:^[42]^

$$*+H{}_{2}O\to *\mathrm{OH}+\left(H{}^{+}+e{}^{-}\right)*\mathrm{OH}\to *O+(H{}^{+}+e{}^{-})*O+H{}_{2}O\to *\mathrm{OOH}+\left(H{}^{+}+e{}^{-}\right)*\mathrm{OOH}\to *+O{}_{2}+\left(H{}^{+}+e{}^{-}\right).$$

Here, we note that alternative mechanistic pathways, such as the lattice oxygen mechanism, have recently been investigated.[^46^, ^47^] However, for a simple estimate of the relative activity of closely related catalyst systems, the energetic comparison of reaction intermediates within the conventional OER mechanism on mechanically (un)strained materials can still serve as a suitable descriptor. Fully consistent with previous work on RuO_2_(110),[^48^, ^49^] these energetics reveal the fourth, oxygen evolution step as the potential‐determining one, requiring the largest minimum thermodynamic overpotential *η*_TD_=0.44 V to make all reaction steps exergonic. In contrast, we compute a much lower overpotential of only *η*_TD_=0.26 V for the coherent 2 ML film of RuO_2_ on TiO_2_. This reduction results exclusively from the strain imposed on RuO_2_ in the core‐shell system, as demonstrated by the almost identical overpotential obtained for an equally strained RuO_2_(110) surface, cf. Figure 5. This strain effectively weakens the O binding that is too strong for the native RuO_2_(110) and that is correspondingly brought closer to the optimum value at the top of the volcano in the epitaxial core‐shell system.^[42]^ Concomitant with this weakening, we also obtain a shift in the potential‐determining step to the final O_2_ evolution, which would then also affect the Tafel slope for such particles. To demonstrate this, we compute the Tafel slope *b* for pristine RuO_2_(110) and the related core‐shell system. It is governed by the apparent transfer coefficient *β*, which specifies the number of electrons transferred counting from the catalyst resting state to the transition state in the free energy diagram[^49^, ^50^] (see the Supporting Information for details):

(4) $$b=\frac{59\;mV/dec}{\beta }.$$

As a result, the computed Tafel slope *b* of RuO_2_(110) is 39.3 mV dec^−1^ at *U*=1.23 V. However, by increasing *U* beyond 1.57 V, the resting state changes from the surface oxo (*O) to the hydroperoxo (*OOH) intermediate, corresponding to a shift of the transition state from the second to the nearest neighbor position in the free energy profile, resulting in a Tafel slope of 118 mV dec^−1^ (see Figure S4). This fully coincides with experimental data under OER conditions.^[51]^ For the core‐shell system, the resting state is given by the surface hydroxo (*OH) intermediate at the OER equilibrium potential (*η*=0). The transition state is the third electron transfer step following the (*OH) state. The Tafel slope thus changes twice with increasing *U*, from *b*=23.6 to 39.3 and finally to 118 mV dec^−1^ by consecutively moving the transition and resting states closer to each other (see Figure S5). Intriguingly, the transition bias between the two Tafel regimes (39.3 and 118 mV dec^−1^) has been lowered for the core‐shell system by more than 0.2 V from *U*=1.57 to 1.35 V. This phenomenon of lowering the transition bias may provide a validation and a guiding principle for the successful design of such core‐shell systems: the lower the transition bias, the better one achieves the desired core‐shell structure. As a further activity descriptor, we adopt the electrochemical‐step symmetry index (ESSI), which was proposed as a measure to quantitatively assess how close a catalyst approaches the catalytically ideal free energy profile:[^52^, ^53^, ^54^]

(5) $$ESSI=\frac{1}{n}\sum_{1}^{n}\left(\frac{\Delta G_{i}^{+}}{e^{-}}-E^{0}\right).$$

Here, $\Delta G_{i}^{+}$ are the reaction energies of those steps in the OER peroxide pathway that are larger than 1.23 eV and *E* ^0^=1.23 V is the OER equilibrium potential. For pristine RuO_2_ the relevant $\Delta G_{i}^{+}$ are the third and fourth steps in Figure 5 (left), that is, ✶OOH formation and O_2_ evolution, and the corresponding ESSI descriptor becomes 0.391 V. For the core‐shell system, the ESSI descriptor yields 0.153 V which is much smaller and indicates that it is significantly closer to the ideal catalyst than pristine RuO_2_. The reduction is, as discussed above, due to the strain induced destabilization of the O binding in the *O state. The equally strained RuO_2_ has an intermediate ESSI value of 0.226 V. In spite of the nearly identical overpotentials *η*_TD_ of the core‐shell and the strained RuO_2_ system, this large ESSI change can simply be attributed to a change in the number of terms n contributing to the average in Equation (5). The second reaction step at the strained RuO_2_ is slightly below (by −0.02 eV) the threshold of 1.23 eV while it is slightly above (+0.08 eV) for the core‐shell system. Such large deviations in the ESSI descriptor by a threshold induced discretization error for the average should be taken with a grain of salt based on typical DFT errors. The simple *η*_TD_ estimate is more robust for such cases. As shown in Figure 5 (right), we can generally conclude though that the RuO_2_/TiO_2_ core‐shell system, especially for the most relevant (011)/(101), (110) and (111) surfaces, could provide a promising RuO_2_‐based OER electrode as the free‐energy change of the second reaction step ranges between 1.03 and 1.43 eV and it exhibits ESSI values below 0.45 V.^[53]^

## Conclusions

Systematically analyzing the interfacial stability, strain and surface free energies, our first‐principles calculations predict a general thermodynamic feasibility of epitaxial core‐shell particles, in which an ultrathin film of RuO_2_ coherently encapsulates a rutile‐structured TiO_2_ core. A high directional dependence of adhesion and lattice mismatch disfavors in particular the (110) orientation, which suffers from maximum strain and offers the smallest density of interfacial bonds. While the same trend with regards to direction is obtained for IrO_2_, its intrinsically higher surface energies render this oxide a less suitable material for such core‐shell concepts.

A key aspect for the practical realization is the ability of both studied MO_2_ to stabilize O‐rich surface terminations at more oxidizing conditions. This increasingly lowers their surface free energies relative to TiO_2_, for which only stoichiometric terminations are found as most stable up to high oxygen chemical potentials. Generally favoring wetting, this points towards growth protocols operating at oxidizing conditions, as for example achievable by electrodeposition. Apart from reducing the precious oxide content, our calculations further indicate two additional benefits of corresponding pseudomorphic core‐shell particles. On the one end, an increased stability at OER operation conditions results as the implied higher surface area and/or exposure of TiO_2_ penalizes a dewetting or strain‐relieving formation of extended defects. On the other end, the additional strain in particular of the prevalent (110) facet leads to a lowered thermodynamic overpotential for few monolayer, coherent RuO_2_ films at rutile TiO_2_. We hope that these insights will stimulate advanced growth endeavors to overcome the presently realized massively loaded composites with their incoherent thick, precious oxide films towards tailored epitaxial core‐shell particles with a predicted increased stability and activity at simultaneously minimized mass loading.

## Methods

In order to assess the interfacial stability we use ab initio thermodynamics^[55]^ to calculate two general key quantities, the interface formation energy $\gamma_{interf}^{\left(hkl\right)}$ and the work of adhesion $W_{adh}^{\left(hkl\right)}$ of a stoichiometric interface with crystallographic orientation (*hkl*). The prior indicates the cost of creating the interface from the respective bulk materials and is defined as

(6) $$\gamma_{interf}^{\left(hkl\right)}=\frac{1}{A^{\left(hkl\right)}}\left\{G_{interf}^{\left(hkl\right)}-\nu_{Ti}^{\left(hkl\right)}G_{{TiO}_{2},bulk}-\nu_{M}^{\left(hkl\right)}G_{{MO}_{2},bulk}\right\}.$$

Here, *A* ^(*hkl*)^ is the area of the interface model employed in the calculations, $G_{interf}^{\left(hkl\right)}$ its Gibbs free energy, $\nu_{Ti}^{\left(hkl\right)}$ its number of Ti atoms, and $\nu_{M}^{\left(hkl\right)}$ its number of M=Ir or Ru atoms. *G* ${}_{\mathrm{TiO}{}_{2},\mathrm{bulk}}$ is the Gibbs free energy per TiO_2_ formula unit of a rutile bulk unit‐cell and *G* ${}_{\mathrm{MO}{}_{2},\mathrm{bulk}}$ correspondingly the Gibbs free energy per MO_2_ formula unit of a rutile bulk unit‐cell. The work of adhesion is the reversible work required to separate the interface into two free surfaces in vacuum

(7) $$W_{adh}^{\left(hkl\right)}=\frac{1}{A^{\left(hkl\right)}}\left\{G_{interf}^{\left(hkl\right)}-G_{{TiO}_{2},surf}^{\left(hkl\right)}-G_{{MO}_{2},surf}^{\left(hkl\right)}\right\},$$

where $G_{{TiO}_{2},surf}^{\left(hkl\right)}$ and $G_{{MO}_{2},surf}^{\left(hkl\right)}$ are the Gibbs free energies of surface models of TiO_2_ and MO_2_, respectively, exhibiting the (*hkl*) facet and the same stoichiometric surface termination as the interface model.

In analogy to $\gamma_{interf}^{\left(hkl\right)}$ , the central quantity determining the thermodynamic stability of a specific facet of a core‐shell particle with termination σ containing $\nu_{M}^{\left(hkl\right),\sigma }$ , $\nu_{O}^{\left(hkl\right),\sigma }$ , and $\nu_{H}^{\left(hkl\right),\sigma }$ with M=(Ir or Ru), O, and H atoms, respectively, in an aqueous environment and under an applied potential U is the surface free energy

(8) $$\gamma_{surf}^{(hkl),\sigma }\left(U\right)=\frac{1}{A^{\left(hkl\right)}}\left\{\begin{array}{c} G_{surf}^{(hkl),\sigma }-\nu_{Ti}^{\left(hkl\right)}G_{{\mathrm{TiO}}_{2},bulk}-\nu_{M}^{(hkl),\sigma }G_{{\mathrm{MO}}_{2},bulk}-\;\\ \left[\nu_{O}^{(hkl),\sigma }-2\left(\nu_{Ti}^{\left(hkl\right)}+\nu_{M}^{(hkl),\sigma }\right)\right]\mu_{H_{2}O}-\;\\ \left[\nu_{H}^{(hkl),\sigma }-2\left(\nu_{O}^{(hkl),\sigma }-2\left(\nu_{Ti}^{\left(hkl\right)}+\nu_{M}^{(hkl),\sigma }\right)\right)\right]\;\\ \left(\mu_{H^{+}}^{aq.}+\mu_{e^{-}}\right)\; \end{array}\right\}$$

Here, the second line accounts for any off‐stoichiometries of the surface termination σ by releasing or taking water molecules from the water environment represented by the chemical potential of water *μ* ${}_{H{}_{2}O}$ at normal conditions. As a byproduct, the surface may be (de)protonated. At the metallic surface, we assume this to proceed in form of a proton coupled electron transfer, thus introducing as relevant reservoir the sum $\left(\mu_{H^{+}}^{aq.}+\mu_{e^{-}}\right)$ of the electrochemical potential of a solvated proton and the electron electrochemical potential in the system. Within the computational hydrogen electrode (CHE) concept of Nørskov and coworkers^[56]^ $\left(\mu_{H^{+}}^{aq.}+\mu_{e^{-}}\right)=\frac{1}{2}\mu_{H_{2}}+eU\;$ , where *μ* ${}_{H{}_{2}}$ is the chemical potential of hydrogen gas at normal conditions and the applied potential U is referenced to the reversible hydrogen electrode (RHE). Here, any treatment of aqueous surrounding is not included in our model. We note that both the solvent dielectric environment and specific interactions with surrounding water molecules can lead to different absolute energetics.[^57^, ^58^] In contrast, we assume such solvent effects to be small when comparing the energetics of materials within the same class differing due to mechanical modifications.

When instead focusing on the stability of a facet in an oxygen gas environment, the surface free energy is analogously given as

(9) $$\gamma_{surf}^{\left(hkl\right),\sigma }\left(\Delta \mu_{O}\right)=\;\frac{1}{A^{\left(hkl\right)}}\left\{\begin{array}{c} G_{surf}^{\left(hkl\right),\sigma }-\nu_{Ti}^{\left(hkl\right)}G_{{TiO}_{2},bulk}-\nu_{M}^{\left(hkl\right),\sigma }G_{{MO}_{2},bulk}-\\ \left[\nu_{O}^{\left(hkl\right),\sigma }-2\left(\nu_{Ti}^{\left(hkl\right)}+\nu_{M}^{\left(hkl\right),\sigma }\right)\right]\left(\frac{1}{2}E_{O_{2}}+\Delta \mu_{O}\right) \end{array}\right\},$$

where the relative chemical potential of oxygen, Δ*μ*_O_=Δ*μ*_O_(*T, p*), now summarizes the dependence of the surface free energy on temperature *T* and oxygen pressure *p*.^[59]^ In detail, as the O_2_ environment forms an ideal‐gas‐like reservoir, the chemical potential of oxygen, *μ*_O_, is expressed as

(10) $$\mu_{O}\left(T,p\right)=\mu_{O}\left(T,p^{\circ }\right)+\frac{1}{2}kTln\left(\frac{p}{p^{\circ }}\right).$$

The chemical potentials of oxygen at the standard state pressure are obtained from reported experiments.^[60]^ Note that both Equations (8) and (9) can also be used for the calculation of the native oxide surfaces ($\nu_{Ti}^{\left(hkl\right)}=0$ and using the $G_{surf}^{\left(hkl\right),\sigma }$ from a corresponding oxide slab model), as well as for the calculation of the Gibbs free energy change of a reaction step $\Delta G=A^{\left(hkl\right)}\left(\gamma_{surf1}^{\left(hkl\right),\sigma }\left(\Delta \mu_{O}\right)-\gamma_{surf2}^{\left(hkl\right),\sigma }\left(\Delta \mu_{O}\right)\right)$ where the two surface terminations surf1 and surf2 differ in their composition according to the reaction step studied.

For the differences of solid‐state Gibbs free energies entering Equations (6)–(9) we follow the approach of Reuter and Scheffler^[59]^ and approximate them with the difference of the corresponding zero‐point energy (ZPE)‐corrected^[61]^ total energy contributions. These total energies are then obtained by first‐principles density‐functional theory (DFT) calculations with the FHI‐aims code^[62]^ and within the generalized‐gradient approximation using the Perdew‐Burke‐Ernzerhof (PBE) functional.^[63]^ Here, we note that it is well established that the PBE level of theory provides an adequate description for the metallic rutile oxides, for example, the reported better agreement of the density‐of‐states of IrO_2_ with experimental XPS spectra compared to hybrid functionals,^[64]^ and we thus use the PBE for IrO_2_ and RuO_2_. ZPE and entropic contributions to the molecular chemical potentials *μ* ${}_{H{}_{2}O}$ and *μ* ${}_{H{}_{2}}$ were obtained from experimental data and reference tables.[^65^, ^66^] To achieve a more accurate electronic structure of the metal‐insulator interface system, Hubbard‐corrected DFT has been applied to the Ti 3d states using an effective on‐site parameter of 4.5 eV.^[67]^ Double counting in this DFT+*U* approach has been treated in the fully localized limit.^[67]^ A detailed account of the computational settings employed in the DFT calculations is provided in the Supporting Information.

## Conflict of interest

The authors declare no conflict of interest.

1

## Supporting information

As a service to our authors and readers, this journal provides supporting information supplied by the authors. Such materials are peer reviewed and may be re‐organized for online delivery, but are not copy‐edited or typeset. Technical support issues arising from supporting information (other than missing files) should be addressed to the authors.

Supporting Information

## Data Availability

The data that support the findings of this study are available in the supplementary material of this article.
