# Supplementary material for: Epitaxial Core‐Shell Oxide Nanoparticles: First‐Principles Evidence for Increased Activity and Stability of Rutile Catalysts for Acidic Oxygen Evolution
Source: ChemSusChem. 2022 Apr 13;15(10):e202200015. doi: 10.1002/cssc.202200015 (PMC9321688; doi:10.1002/cssc.202200015)
Supplement: Supplementary file 1 — Supporting Information [file CSSC-15-0-s001.pdf]

ChemSusChem

Supporting Information

Epitaxial Core-Shell Oxide Nanoparticles: First-Principles Evidence for Increased Activity and Stability of Rutile Catalysts for Acidic Oxygen Evolution

Yonghyuk Lee, Christoph Scheurer,* and Karsten Reuter© 2022 The Authors. ChemSusChem published by Wiley-VCH GmbH. This is an open access article under the terms of the Creative Commons Attribution License, which permits use, distribution and reproduction in any medium, provided the original work is properly cited.

I. COMPUTATIONAL SETTINGS

All density-functional theory (DFT) calculations have been performed using the numeric atom-centered basis all-electron code FHI-AIMS [1] and the semi-local generalized gradient approximation (GGA) due to Perdew-Burke-Ernzerhof (PBE) [2] as the electronic exchange and correlation (xc) functional with default light integration settings, and standard tier1 (Ir, Ru, Ti) and tier2 (O, H) basis sets. To achieve a more accurate electronic structure of the metal-insulator interface system, an effective Hubbard U is applied at the localized Ti $3d$ states with the fully localized limit approximation for the double counting correction functional [3]. The absolute value of the Hubbard $U = 4.5$ eV is chosen as the one with which the calculated electronic band gap becomes closest to the experimentally measured value before the band structure gets corrupted [3]. The Brillouin-zone integration is carried out on a grid of k-points with reciprocal distances of 0.02 Å^{-1}, producing k-grids of dimension $11 \times 11 \times 16$ (with 969 irreducible k-points) for bulk rutile IrO_2 and RuO_2, and $11 \times 11 \times 17$ (with 1029 irreducible k-points) for TiO_2. The same grid density is applied for all slab models and the resulting k-grids are listed in Table S1. We iteratively minimize lattice stress and internal atomic displacements until the maximum residual force component per atom is lower than 10^{-2} eV/Å, in order to obtain the relaxed unit cell structure of bulk IrO_2, RuO_2, and TiO_2.

Table S1. Used k-grids and corresponding irreducible k-points of all (1×1) slab models for IrO_2, RuO_2 and TiO_2. Additionally shown is the number of slab layers employed for each orientation (and considering a stoichiometric termination).

	IrO_2		RuO_2		TiO_2		Slab thickness
	k-grid	irreducible	k-grid	irreducible	k-grid	irreducible	
(001)	$(11 \times 11 \times 1)$	61	$(11 \times 11 \times 1)$	61	$(11 \times 11 \times 1)$	61	13
(010)/(100)	$(11 \times 16 \times 1)$	89	$(11 \times 16 \times 1)$	89	$(11 \times 17 \times 1)$	94	13
(011)/(101)	$(11 \times 10 \times 1)$	56	$(11 \times 10 \times 1)$	56	$(11 \times 10 \times 1)$	56	7
(110)	$(16 \times 8 \times 1)$	66	$(16 \times 8 \times 1)$	66	$(17 \times 8 \times 1)$	69	7
(111)	$(10 \times 12 \times 1)$	62	$(10 \times 12 \times 1)$	62	$(10 \times 12 \times 1)$	62	13

The specific number of slab layers employed for the calculations of the five symmetry-inequivalent low index surfaces (001), (010)/(100), (011)/(101), (110) and (111) of the native oxides are summarized in Table S1. At these thicknesses and a minimum vacuum distance

of 20 Å to decouple periodic slabs, the surface free energies of the slabs are converged to within ±5 meV/Å^2. (1 × 1) surface unit cells are employed for the rhombus-like surfaces ($a \simeq b$), (001), (011)/(101) and (111), while (2×1) supercells are used for the rectangular (010)/(100) and (110) surfaces. Different terminations are considered for every slab model at each low index surface, including -O, -OH, -OOH, -OH$_2$, -OO, following the protocol established in ref. 4. Furthermore, additional structures with surface vacancies are included. Since the potential energy surface of adsorbed -OH and -OOH groups features many shallow energy minima, various initial configurations of these intermediates have been considered in the geometry relaxations to find the most stable structures. In total, up to 100 models for each surface system are included in our initial screening approach. The collection of relaxed surface structures is subsequently reduced to the most relevant surfaces in thermodynamic equilibrium and at the applied potential.

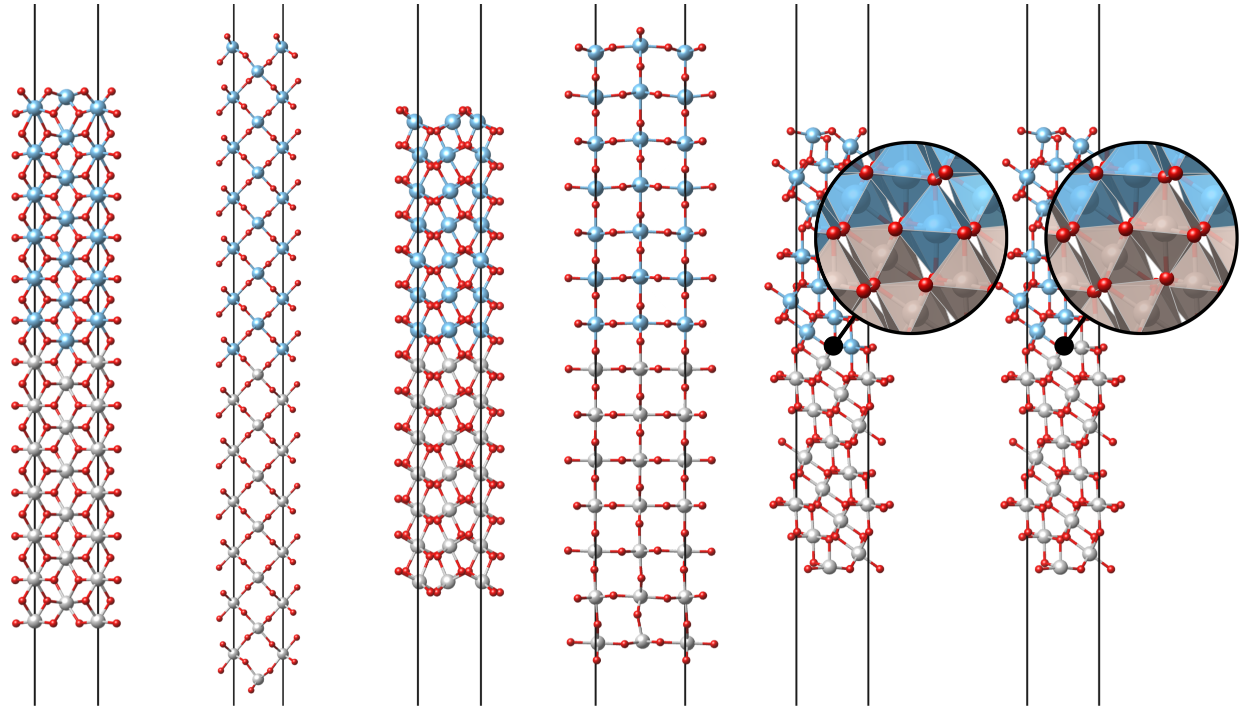

Figure S1. Atomic structures of coherent stoichiometric MO_2/TiO_2 interface models for all five symmetry-inequivalent low-index orientations. From left to right, (001), (010)/(100), (011)/(101), (110), (111)-t1 and (111)-t2 are shown. Ti, M (M=Ir or Ru) and O atoms are represented in blue, gray and red spheres, respectively.

Atomic geometries of stoichiometric coherent MO_2/TiO_2 (M=Ir or Ru) interfaces for all five low-index orientations are illustrated in Fig. S1. As discussed in the main text, we use the optimized TiO_2 bulk lattice constants to eliminate the increasing strain in the coherent

MO2 film of increasing thickness when calculating the interface specific properties. The interface structures consist of two stoichiometric surfaces, one for TiO_2 and one for strained MO_2 at each end, and their interface in the middle. All models are twice as thick as the separated slab models which we use for all pure surface calculations, to decouple the possible interactions between surface and interface. As in the native oxide surface calculations, a 20 Å vacuum separates the periodic slabs. In order to minimize the strain applied to the MO_2 layers, all atoms are fully relaxed without constrained region. In the case of (111), two stoichiometric interfaces are possible as shown in Fig. S1, which are named as (111)-t1 and t2.

Table S2. Optimized DFT-PBE lattice constants a_0 and c_0 of bulk-rutile IrO_2, RuO_2 and TiO_2. $a^{(hkl)}$ and $b^{(hkl)}$ are the two unit-cell lattice vectors that are parallel to the corresponding low-index (hkl) surface, and R_{MO_2/TiO_2} is the corresponding relative lattice mismatch to TiO_2.

	IrO_2 (Å)	RuO_2 (Å)	TiO_2 (Å)	R_{IrO_2/TiO_2} (%)	R_{RuO_2/TiO_2} (%)
a_0	4.529	4.527	4.578	-1.1	-1.1
c_0	3.181	3.122	2.955	7.6	5.6
$a^{(001)}$	4.529	4.527	4.578	-1.1	-1.1
$b^{(001)}$	4.529	4.527	4.578	-1.1	-1.1
$a^{(010)/(100)}$	4.529	4.527	4.578	-1.1	-1.1
$b^{(010)/(100)}$	3.181	3.122	2.955	7.6	5.6
$a^{(011)/(101)}$	4.529	4.527	4.578	-1.1	-1.1
$b^{(011)/(101)}$	5.534	5.499	5.449	1.6	0.9
$a^{(110)}$	3.181	3.122	2.955	7.6	5.6
$b^{(110)}$	6.405	6.403	6.475	-1.1	-1.1
$a^{(111)}$	6.405	6.403	6.475	-1.1	-1.1
$b^{(111)}$	5.534	5.499	5.449	1.6	0.9

Table S3. Calculated surface free energies $\gamma_{\text{surf}}^{(hkl),\sigma}$ (in meV/Å^2) for all five symmetry-inequivalent low-index orientations (hkl) of pure IrO_2, RuO_2 and TiO_2, and core-shell IrO_2/TiO_2 and RuO_2/TiO_2 with 1-3 ML thickness shell at the applied potential U of 1.23 V.

	$\gamma_{\text{surf}}^{(hkl),\sigma}$ (IrO_2)	$\gamma_{\text{surf}}^{(hkl),\sigma}$ (RuO_2)	$\gamma_{\text{surf}}^{(hkl),\sigma}$ (TiO_2)	$\gamma_{\text{surf}}^{(hkl),\sigma}$ (IrO_2/TiO_2)			$\gamma_{\text{surf}}^{(hkl),\sigma}$ (RuO_2/TiO_2)		
				1 ML	2 ML	3 ML	1 ML	2 ML	3 ML
(001)	32	10	64	59	68	69	39	33	33
(010)/(100)	34	34	39	63	76	94	26	58	60
(011)/(101)	24	14	40	38	37	39	29	32	31
(110)	25	26	30	67	79	117	45	57	68
(111)	19	6	95	36	35	37	25	19	24

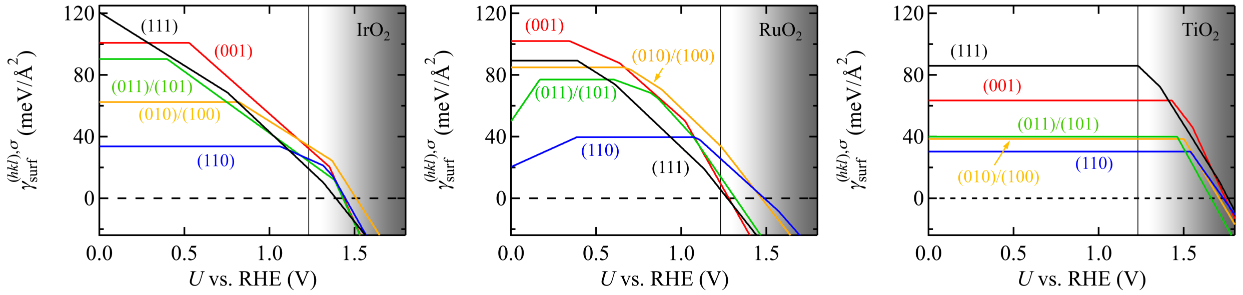

Figure S2. The lowest surface free energies $\gamma_{\text{surf}}^{(hkl),\sigma}$ for all five low-index facets (hkl) of pure rutile IrO_2 (left panel), RuO_2 (center panel) and TiO_2 (right panel) in aqueous environment as a function of the applied potential U from open-circuit ($U = 0$ V vs. RHE) to PEM operating conditions. The vertical black line indicates the OER equilibrium potential $U = 1.23$ V.

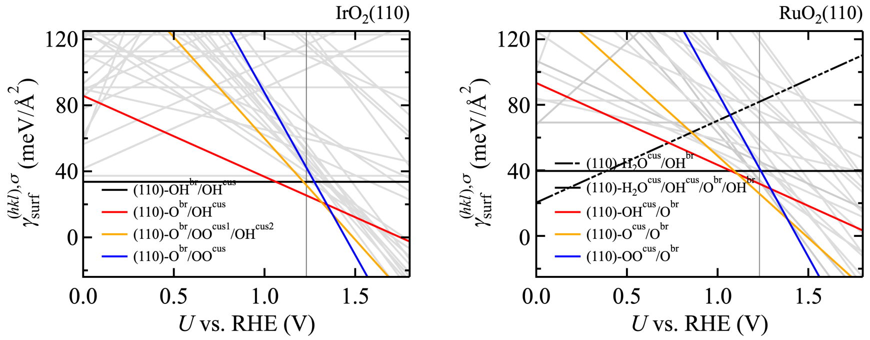

Figure S3. Computed surface free energies $\gamma_{\text{surf}}^{(hkl),\sigma}$ for the (110) facet of IrO_2 (left panel) and RuO_2 (right panel) as a function of the applied potential U from open-circuit ($U = 0$ V vs. RHE) to PEM operating conditions. Thermodynamically less favorable surface phases are indicated by pale gray lines. More hydrogenated surfaces are overall the most stable at open-circuit (0 V vs. RHE) and surfaces are gradually deprotonated as potential increases. Eventually at OER-active conditions, surfaces are completely deprotonated or exhibit superoxo species as detailed in ref. 4.

Table S4. Calculated critical potentials U_{critical} (in V) for all five symmetry-inequivalent low-index orientations (hkl) of pure IrO_2 and RuO_2, and core-shell IrO_2/TiO_2 and RuO_2/TiO_2 with a 2 ML thick shell.

	$U^{\mathrm{IrO_2}}_{\mathrm{critical}}$	$U^{\mathrm{RuO_2}}_{\mathrm{critical}}$	$U^{\mathrm{IrO_2/TiO_2}}_{\mathrm{critical}}$	$U^{\mathrm{RuO_2/TiO_2}}_{\mathrm{critical}}$
(001)	1.44	1.28	1.52	1.40
(010)/(100)	1.51	1.47	1.80	1.62
(011)/(101)	1.43	1.32	1.62	1.43
(110)	1.45	1.49	1.69	1.68
(111)	1.39	1.27	1.50	1.36

II. RENUMBERING OF THE ELECTRON-TRANSFER STEPS AND TAFEL SLOPE

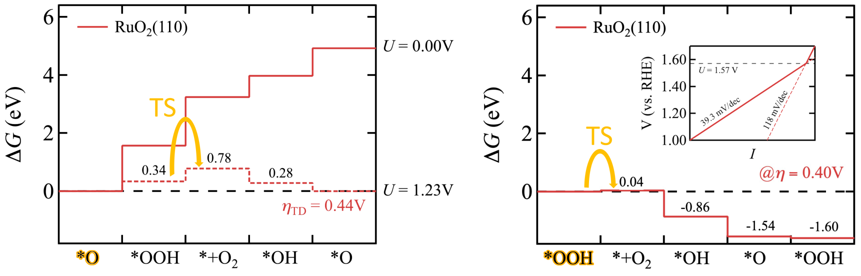

Figure S4. Gibbs free energy change profile ΔG for pristine $RuO_2(110)$ after renumbering of the electron-transfer steps at $U = 1.23$ V ($\eta = 0.00$ V) (left panel) and $U = 1.63$ V ($\eta = 0.40$ V) (right panel). ΔG for each reaction intermediate as well as the thermodynamic overpotential η_{TD} are remarked. The catalyst resting state is highlighted in the x-axis label as *O and *OOH, respectively. Along with the change in the resting state, the transition state (TS) shifts from the second to the first step in the free energy profile and the Tafel slope (inset of the right panel) bends off at $U = 1.57$ V.

In a number of studies [5–9], the OER peroxide pathway is considered to be initiated from the catalytically active site (*) without any adsorbates. However, the most stable intermediate termination varies at the given applied bias, and the OER should be initiated from the intermediate, which is called the catalyst resting state. As shown in Fig. 5, the resting state of $RuO_2(110)$ at the OER equilibrium potential ($U = 1.23$ V) is the surface oxo (*O). Based on this, the updated free energy profile after the renumbering of the electron-transfer steps is illustrated in Fig. S4 (left). Here, the transition state (TS) is located at the second electron transfer step. As we increase the overpotential (η) to 0.34 V, ΔG for *O and *OOH become identical and *O/*OOH mixed phase may be stabilized. The overpotential above 0.34 V, the resting state switches from the *O to the *OOH, which again requires renumbering of the steps. Fig. S4 (right) shows the free energy profile at $\eta = 0.40$ V and the transition state shifts to the first electron transfer step. The computed Tafel plot (see the inset of Fig. S4 (right)) indicates that the Tafel slope shifts from 39.3 to 118 mV/dec at $U = 1.57$ V.

The same procedure has been applied to the core-shell and shown in Fig. S5. In contrast to $RuO_2(110)$, the surface hydroxo (*OH) is the catalyst resting state at the OER equilibrium potential, and the transition state is the third electron transfer step. The catalyst resting state thus switches twice from *OH to *O to *OOH, as increasing the overpotential to

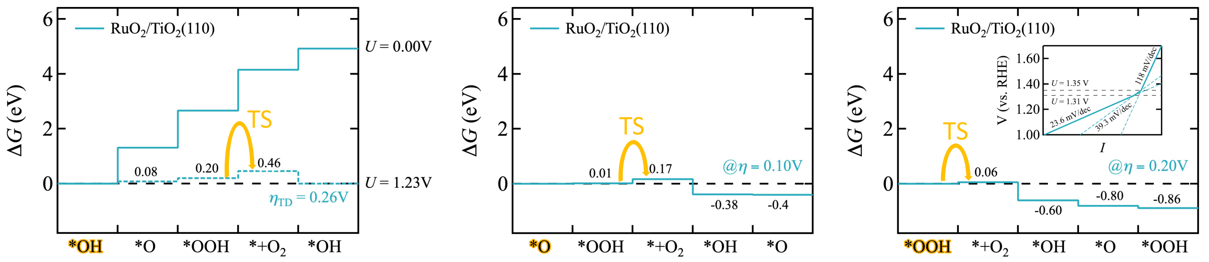

Figure S5. Gibbs free energy change profile ΔG for core-shell(110) after renumbering of the electron-transfer steps at $U = 1.23$ V ($\eta = 0.00$ V) (left panel), $U = 1.33$ V ($\eta = 0.10$ V) (middle panel) and $U = 1.43$ V ($\eta = 0.20$ V) (right panel). ΔG for each reaction intermediate as well as the thermodynamic overpotential η_{TD} are remarked. The catalyst resting state is highlighted in the x-axis label as *OH, *O, and *OOH, respectively. Different from the pristine RuO_2 in Fig. S4, the transition state (TS) shifts twice from the third to the second and eventually to the first step. The corresponding Tafel slope (inset of the right panel) bends from 23.6 to 39.3 to 118 mv/dec at $U = 1.31$ and 1.35 V, respectively.

0.08 and to 0.12 V, respectively, along with the transition state shifting to the second and eventually to the first step. The corresponding Tafel slope changes from 23.6 to 39.3 to 118 mV/dec as shown in the inset of Fig. S5 (right).

Table S5. Calculated atomic coordinates of the most stable $RuO_2/TiO_2(001)$ at U =1.23 V in the FHI-AIMS geometry.in format.

lattice_vector	4.57828842	0.00000000	0.00000000	
lattice_vector	0.00000000	4.57828842	0.00000000	
lattice_vector	0.00000000	0.00000000	46.59747986	
atom	3.26884429	3.26884639	36.56335692	O
atom	1.30939530	1.30939384	36.56330977	O
atom	3.59257421	0.98571062	35.18838101	O
atom	0.98570817	3.59257250	35.18838076	O
atom	2.28915566	2.28915599	35.57074423	Ru
atom	3.19028604	3.19028633	33.76609660	O
atom	1.38801658	1.38801688	33.76610640	O
atom	0.00000557	0.00000612	33.86042630	Ru
atom	2.28914716	2.28914747	32.28934946	Ru
atom	3.69030340	0.88798690	32.29561541	O
atom	0.88798700	3.69030416	32.29561562	O
atom	0.00000047	0.00000057	30.78008375	Ru
atom	1.36614731	1.36614872	30.76058144	O
atom	3.21214222	3.21214106	30.76057786	O
atom	3.65780700	0.92047788	29.23511881	O
atom	0.92048098	3.65781000	29.23511885	O
atom	2.28914416	2.28914411	29.22015814	Ti
atom	3.18529025	3.18528783	27.75698688	O
atom	1.39299756	1.39299989	27.75698694	O
atom	0.00000005	0.00000001	27.77797800	Ti
atom	3.68586097	0.89242745	26.25401547	O
atom	0.89242745	3.68586097	26.25401547	O
atom	2.28914421	2.28914421	26.25401547	Ti
atom	3.18157166	3.18157166	24.77637770	O
atom	1.39671676	1.39671676	24.77637770	O
atom	0.00000000	0.00000000	24.77637770	Ti
atom	2.28914421	2.28914421	23.29873993	Ti
atom	0.89242745	3.68586097	23.29873993	O
atom	3.68586097	0.89242745	23.29873993	O
atom	3.18157166	3.18157166	21.82110216	O
atom	0.00000000	0.00000000	21.82110216	Ti
atom	1.39671676	1.39671676	21.82110216	O
atom	3.68586097	0.89242745	20.34346439	O
atom	0.89242745	3.68586097	20.34346439	O
atom	2.28914421	2.28914421	20.34346439	Ti
atom	3.18529169	3.18528962	18.84049967	O
atom	1.39299678	1.39299899	18.84049934	O
atom	-0.00000014	-0.00000010	18.81950703	Ti
atom	2.28914394	2.28914404	17.37732469	Ti
atom	3.65780914	0.92047665	17.36235964	O
atom	0.92047922	3.65781193	17.36235960	O
atom	-0.00000115	-0.00000097	15.81739347	Ru
atom	1.36614441	1.36614565	15.83691901	O
atom	3.21214146	3.21214052	15.83691469	O
atom	3.69030328	0.88798242	14.30185452	O
atom	0.88798261	3.69030378	14.30185460	O
atom	2.28914028	2.28914043	14.30814760	Ru
atom	3.19027274	3.19027308	12.83140198	O
atom	1.38799804	1.38799832	12.83141233	O
atom	-0.00000763	-0.00000753	12.73703392	Ru
atom	3.59257539	0.98571951	11.40908019	O
atom	0.98571716	3.59257353	11.40907999	O
atom	2.28912902	2.28912930	11.02677643	Ru
atom	3.26887261	3.26887505	10.03419344	O
atom	1.30946729	1.30946615	10.03414142	O

Table S6. Calculated atomic coordinates of the most stable $RuO_2/TiO_2(010)/(100)$ at U =1.23 V in the FHI-AIMS geometry.in format.

lattice_vector	4.57828842	0.00000000	0.00000000	
lattice_vector	0.00000000	2.95527554	0.00000000	
lattice_vector	0.00000000	0.00000000	59.41974088	
atom	1.20860208	-0.00000025	49.84171525	O
atom	3.61946415	1.47763751	48.99433895	O
atom	0.17267637	-0.00000026	48.51592659	Ru
atom	0.95222979	1.47763750	47.42678090	O
atom	3.25805729	-0.00000030	46.82384714	O
atom	2.31803023	1.47763746	45.98891371	Ru
atom	1.35217785	-0.00000029	45.08493608	O
atom	3.66402882	1.47763757	44.51120849	O
atom	0.00390559	-0.00000014	43.60780055	Ti
atom	0.91628284	1.47763760	42.73681003	O
atom	3.19208054	-0.00000012	42.15046425	O
atom	2.28884495	1.47763767	41.26190615	Ti
atom	1.34872473	-0.00000010	40.37619055	O
atom	3.64013154	1.47763771	39.81109968	O
atom	-0.00581259	-0.00000005	38.92399563	Ti
atom	0.89286172	1.47763773	38.02630597	O
atom	3.18157166	0.00000000	37.46973052	O
atom	2.28914421	1.47763777	36.57730307	Ti
atom	1.39671676	0.00000000	35.68487562	O
atom	3.68586097	1.47763777	35.18058631	O
atom	0.00000000	0.00000000	34.28815886	Ti
atom	0.89242745	1.47763777	33.39573141	O
atom	3.18157166	0.00000000	32.89144210	O
atom	2.28914421	1.47763777	31.99901465	Ti
atom	1.39671676	0.00000000	31.10658720	O
atom	3.68586097	1.47763777	30.60229789	O
atom	0.00000000	0.00000000	29.70987044	Ti
atom	0.89242745	1.47763777	28.81744299	O
atom	3.18157166	0.00000000	28.31315368	O
atom	2.28914421	1.47763777	27.42072623	Ti
atom	1.39671676	0.00000000	26.52829878	O
atom	3.68586097	1.47763777	26.02400947	O
atom	0.00000000	0.00000000	25.13158202	Ti
atom	0.89242745	1.47763777	24.23915457	O
atom	3.18157166	0.00000000	23.73486526	O
atom	2.28914421	1.47763777	22.84243781	Ti
atom	1.39671676	0.00000000	21.95001036	O
atom	3.68542084	1.47763870	21.39340730	O
atom	0.00580924	0.00000131	20.49572078	Ti
atom	0.93815090	1.47763940	19.60861761	O
atom	3.22956041	0.00000209	19.04350407	O
atom	2.28943330	1.47763995	18.15779551	Ti
atom	1.38619288	0.00000256	17.26924215	O
atom	3.66199115	1.47764091	16.68287805	O
atom	-0.00392765	0.00000415	15.81189406	Ti
atom	0.91422942	1.47764097	14.90848436	O
atom	3.22609558	0.00000551	14.33474615	O
atom	2.26022535	1.47764335	13.43078915	Ru
atom	1.32018136	0.00000583	12.59585413	O
atom	3.62599259	1.47764326	11.99291288	O
atom	-0.17274140	0.00000613	10.90379958	Ru
atom	0.95895196	1.47764330	10.42548337	O
atom	3.36971573	0.00000683	9.57759866	O

Table S7. Calculated atomic coordinates of the most stable $RuO_2/TiO_2(011)/(101)$ at U =1.23 V in the FHI-AIMS geometry.in format.

lattice_vector	4.57828842	0.00000000	0.00000000	
lattice_vector	0.00000000	5.44925485	0.00000000	
lattice_vector	0.00000000	0.00000000	49.03765138	
atom	1.23445938	0.97227401	10.81196129	O
atom	1.05453583	3.69692860	10.81196581	O
atom	3.61988426	5.36331930	11.68253740	O
atom	3.24760072	2.63867576	11.68253223	O
atom	0.01122341	3.85123447	12.15520728	Ru
atom	2.27794018	1.12660224	12.15518517	Ru
atom	1.38202586	5.03273124	13.11361174	O
atom	0.90711312	2.30807912	13.11359131	O
atom	3.17096626	1.00184394	14.11100756	O
atom	3.69645870	3.72646088	14.11100895	O
atom	-0.00391658	2.13747921	14.84817528	Ru
atom	2.29306603	4.86211341	14.84818224	Ru
atom	1.36406967	3.25492305	15.63771605	O
atom	0.92506577	0.53028676	15.63771885	O
atom	3.23441041	4.84781831	16.63037325	O
atom	3.63301725	2.12319229	16.63036550	O
atom	2.29354835	3.38983623	17.40160464	Ti
atom	-0.00441065	0.66521079	17.40160619	Ti
atom	1.39068267	1.76547444	18.16381426	O
atom	0.89845333	4.49010020	18.16381257	O
atom	3.19006021	3.28029382	19.12807166	O
atom	3.67736597	0.55566644	19.12807232	O
atom	0.00983776	4.47493246	19.89952571	Ti
atom	2.27930171	1.75030500	19.89952570	Ti
atom	1.39251540	0.18943952	20.66658100	O
atom	0.89662504	2.91406692	20.66658028	O
atom	3.18157166	1.71141142	21.65716008	O
atom	3.68586097	4.43603885	21.65716008	O
atom	0.00000000	2.88488786	22.41463665	Ti
atom	2.28914421	0.16026044	22.41463665	Ti
atom	1.39671676	4.05836430	23.17211322	O
atom	0.89242745	1.33373688	23.17211322	O
atom	3.68586097	2.83331448	24.14008741	O
atom	3.18157166	0.10868706	24.14008741	O
atom	2.28914421	4.00679092	24.89756398	Ti
atom	0.00000000	1.28216350	24.89756398	Ti
atom	1.39671676	2.45563994	25.65504055	O
atom	0.89242745	5.18026736	25.65504055	O
atom	3.18157166	3.95521754	26.62301474	O
atom	3.68586097	1.23059011	26.62301474	O
atom	0.00000000	5.12869398	27.38049131	Ti
atom	2.28914421	2.40406655	27.38049131	Ti
atom	0.89242745	3.57754299	28.13796788	O
atom	1.39671676	0.85291557	28.13796788	O
atom	3.18577218	2.37488822	29.12854985	O
atom	3.68166145	5.09951557	29.12855001	O
atom	2.29898572	0.81402254	29.89560487	Ti
atom	-0.00984032	3.53865012	29.89560494	Ti
atom	1.38822567	4.73329034	30.66706026	O
atom	0.90092049	2.00866301	30.66705988	O
atom	3.18760307	0.79885588	31.63131873	O
atom	3.67983177	3.52348361	31.63131913	O
atom	2.28473794	4.62375093	32.39352802	Ti
atom	0.00440844	1.89912320	32.39352706	Ti
atom	1.34387532	3.16576833	33.16476226	O
atom	0.94527114	0.44114106	33.16476281	O
atom	3.21421536	4.75866720	34.15741866	O
atom	3.65321928	2.03404167	34.15741723	O
atom	0.00392007	0.42684961	34.94695176	Ru
atom	2.28522380	3.15147525	34.94695223	Ru
atom	1.40732197	1.56248787	35.68412049	O
atom	0.88182317	4.28711810	35.68411920	O
atom	3.19625695	2.98086211	36.68153006	O
atom	3.67117178	0.25623964	36.68152767	O
atom	2.30035837	1.43772340	37.63993780	Ru
atom	-0.01121811	4.16235445	37.63993196	Ru
atom	1.33070729	5.37490031	38.11260218	O
atom	0.95843250	2.65027724	38.11259853	O
atom	3.34382964	1.59211133	38.98315234	O
atom	3.52364317	4.31672641	38.98315209	O

Table S8. Calculated atomic coordinates of the most stable $RuO_2/TiO_2(110)$ at U =1.23 V in the FHI-AIMS geometry.in format.

lattice_vector	2.95527554	0.00000000	0.00000000	
lattice_vector	0.00000000	6.47467758	0.00000000	
lattice_vector	0.00000000	0.00000000	56.32389945	
atom	0.00006491	0.00014440	46.41716379	O
atom	0.00008980	3.23747397	45.69062322	O
atom	0.00011984	0.00018422	44.71730337	Ru
atom	1.47773480	5.20274024	44.50814301	O
atom	1.47773478	1.27229512	44.50814778	O
atom	1.47771372	3.23753002	44.52960106	Ru
atom	0.00006459	3.23748277	43.17536725	O
atom	-0.00000743	0.00011629	42.65545754	O
atom	-0.00000138	3.23742013	41.25376539	Ru
atom	1.47763718	4.53517403	41.24473364	O
atom	1.47763717	1.93967283	41.24475497	O
atom	1.47762518	0.00008411	41.30730385	Ru
atom	-0.00001199	0.00009152	39.96235528	O
atom	0.00002035	3.23739738	39.24437889	O
atom	1.47772438	3.23740927	37.95099239	Ti
atom	1.47767170	1.26522538	37.98142316	O
atom	0.00000124	0.00007160	37.97417752	Ti
atom	1.47767171	5.20958440	37.98144150	O
atom	0.00004365	3.23740973	36.67705556	O
atom	0.00005226	0.00006393	35.96592732	O
atom	0.00006438	3.23741675	34.66727200	Ti
atom	1.47770514	4.49932766	34.67254732	O
atom	1.47770431	1.97547727	34.67256922	O
atom	1.47771491	0.00008764	34.68472756	Ti
atom	0.00005088	0.00005468	33.40177707	O
atom	0.00000000	3.23733879	32.66137152	O
atom	0.00000000	0.00000000	31.39928851	Ti
atom	1.47763777	5.21259457	31.39928851	O
atom	1.47763777	1.26208300	31.39928851	O
atom	1.47763777	3.23733879	31.39928851	Ti
atom	0.00000000	3.23733879	30.13720551	O
atom	0.00000000	0.00000000	29.42403273	O
atom	1.47763777	0.00000000	28.16194973	Ti
atom	1.47763777	1.97525579	28.16194973	O
atom	1.47763777	4.49942179	28.16194973	O
atom	0.00000000	3.23733879	28.16194973	Ti
atom	0.00000000	0.00000000	26.89986673	O
atom	0.00000000	3.23733879	26.18669394	O
atom	1.47763777	3.23733879	24.92461094	Ti
atom	0.00000000	0.00000000	24.92461094	Ti
atom	1.47763777	5.21259457	24.92461094	O
atom	1.47763777	1.26208300	24.92461094	O
atom	0.00000000	3.23733879	23.66252794	O
atom	-0.00001962	0.00000058	22.92184406	O
atom	-0.00001351	3.23732950	21.65579893	Ti
atom	1.47763550	4.49931351	21.65051574	O
atom	1.47763767	1.97535847	21.65052317	O
atom	1.47760231	-0.00000345	21.63884047	Ti
atom	-0.00002532	0.00000290	20.35743158	O
atom	-0.00002120	3.23732567	19.64567615	O
atom	1.47761693	3.23732918	18.37163468	Ti
atom	1.47761375	5.20948383	18.34171474	O
atom	-0.00002322	-0.00000752	18.34937511	Ti
atom	1.47761373	1.26517523	18.34171048	O
atom	-0.00002315	3.23732693	17.07828606	O
atom	-0.00002567	-0.00000623	16.36125164	O
atom	-0.00002486	3.23732892	15.06885995	Ru
atom	1.47761280	4.53519837	15.07820572	O
atom	1.47761269	1.93946086	15.07820260	O
atom	1.47761253	-0.00000906	15.01663973	Ru
atom	-0.00002460	-0.00001096	13.66880312	O
atom	-0.00002504	3.23733166	13.14707014	O
atom	-0.00002304	-0.00001023	11.60695495	Ru
atom	1.47761415	5.20277652	11.81616225	O
atom	1.47761416	1.27188229	11.81616609	O
atom	1.47761371	3.23733016	11.79346677	Ru
atom	-0.00002390	3.23733444	10.63238670	O
atom	-0.00002374	-0.00000972	9.90708429	O

Table S9. Calculated atomic coordinates of the most stable $RuO_2/TiO_2(111)$ at U =1.23 V in the FHI-AIMS geometry.in format.

lattice_vector	5.44925485	0.00000000	0.00000000	
lattice_vector	1.60272437	5.20822935	0.00000000	
lattice_vector	0.00000000	0.00000000	40.97524937	
atom	2.49305173	1.84252522	9.03052398	O
atom	4.26545034	3.15185131	10.80196379	O
atom	2.76372365	2.04256661	10.69485294	Ru
atom	1.60836041	3.50957675	11.03546367	O
atom	3.82629111	0.50696458	11.03573042	O
atom	1.28990428	0.95386862	11.30830197	O
atom	5.49031271	4.05639891	11.95315891	Ru
atom	2.69597620	1.99226547	13.05342867	O
atom	1.15715739	0.85573629	13.16018625	Ru
atom	3.91706571	4.45469963	13.19001471	O
atom	5.40861690	2.43491907	13.18962449	O
atom	6.60357054	4.87776546	13.37157126	O
atom	3.82663897	2.82695839	14.28958051	Ru
atom	1.05379805	0.77899794	15.20504841	O
atom	2.22231494	3.25193640	15.42051400	O
atom	3.76129193	1.16817991	15.42015103	O
atom	6.59569077	4.87146647	15.38767794	Ti
atom	4.95449396	3.65945355	15.66679618	O
atom	2.25390895	1.66484021	16.53305418	Ti
atom	6.51508224	4.81204356	17.35022959	O
atom	4.96843354	3.66942962	17.63915111	Ti
atom	2.19223273	0.05009563	17.63915111	O
atom	0.69265513	2.08053426	17.63915111	O
atom	3.41728255	2.52382923	17.87956175	O
atom	0.64108175	0.47347003	18.73045841	Ti
atom	4.91686015	3.63134017	19.58135508	O
atom	3.36570917	2.48573979	19.82176572	Ti
atom	2.19223273	4.07463515	19.82176572	O
atom	4.53918561	0.89684442	19.82176572	O
atom	1.81455819	1.34013940	20.06217635	O
atom	6.09033659	4.49800955	20.91307302	Ti
atom	3.31413579	2.44765034	21.76396969	O
atom	1.76298481	1.30204995	22.00438032	Ti
atom	6.03876321	2.89094532	22.00438032	O
atom	4.53918561	4.92138394	22.00438032	O
atom	0.21183382	0.15644957	22.24479095	O
atom	4.48761223	3.31431971	23.09568762	Ti
atom	1.71141142	1.26396050	23.94658429	O
atom	0.16026044	0.11836012	24.18699492	Ti
atom	2.93646124	3.73769411	24.18699492	O
atom	4.43603885	1.70725548	24.18699492	O
atom	5.66559053	4.18397515	24.47591636	O
atom	2.87478439	2.12294904	25.29309168	Ti
atom	0.17419943	0.12833574	26.15934948	O
atom	1.36740154	2.61960906	26.40599610	O
atom	5.58498152	4.12455191	26.43846832	Ti
atom	2.90637814	0.53585301	26.40563306	O
atom	4.07489481	3.00879082	26.62109849	O
atom	1.30205388	0.96083050	27.53656645	Ru
atom	5.57710148	4.11825259	28.45457482	O
atom	5.16933064	1.35286942	28.63652206	O
atom	2.81435128	4.54131848	28.63613179	O
atom	3.97153500	2.93205215	28.66596057	Ru
atom	2.43271594	1.79552257	28.77272014	O
atom	6.69035899	4.93961876	29.87298821	Ru
atom	3.83878855	2.83392012	30.51784465	O
atom	3.52033203	0.27821121	30.79068312	O
atom	2.36496862	1.74522144	31.13129300	Ru
atom	1.30240123	3.28082376	30.79041628	O
atom	0.86324193	0.63593676	31.02418302	O
atom	2.63564115	1.94526332	32.79562190	O

[1] V. Blum, R. Gehrke, F. Hanke, P. Havu, V. Havu, X. Ren, K. Reuter, M. Scheffler. *Comput. Phys. Commun.* **2009**, *180*, 2175–2196.

[2] J. P. Perdew, K. Burke, M. Ernzerhof. *Phys. Rev. Lett.* **1996**, *77*, 3865.

[3] M. Kick, K. Reuter, H. Oberhofer. *J. Chem. Theory Comput.* **2019**, *15*, 1705–1718.

[4] D. Opalka, C. Scheurer, K. Reuter. *ACS Catalysis* **2019**, *9*, 4944–4950.

[5] J. Rossmeisl, Z.-W. Qu, H. Zhu, G.-J. Kroes, J. K. Nørskov. *J. Electroanal. Chem.* **2007**, *607*, 83–89.

[6] I. C. Man, H.-Y. Su, F. Calle-Vallejo, H. A. Hansen, J. I. Martínez, N. G. Inoglu, J. Kitchin, T. F. Jaramillo, J. K. Nørskov, J. Rossmeisl. *ChemCatChem* **2011**, *3*, 1159–1165.

[7] N. B. Halck, V. Petrykin, P. Krtil, J. Rossmeisl. *Phys. Chem. Chem. Phys.* **2014**, *16*, 13682–13688.

[8] J. A. Gauthier, C. F. Dickens, L. D. Chen, A. D. Doyle, J. K. Nørskov. *J. Phys. Chem. C* **2017**, *121*, 11455–11463.

[9] A. F. Pedersen, M. Escudero-Escribano, B. Sebok, A. Bodin, E. Paoli, R. Frydendal, D. Friebel, I. E. L. Stephens, J. Rossmeisl, I. Chorkendorff, A. Nilsson. *J. Phys. Chem. B* **2018**, *122*, 878–887.
